# Concurrent Germline-Somatic Alterations and Associations with Cancer Outcomes: A Systematic Review of Concurrent Data Use

**DOI:** 10.21203/rs.3.rs-8296996/v1

**Published:** 2026-02-02

**Authors:** Lipika R. Pal, Alejandro A. Schäffer, Santiago Avila, Jonathan Wooten, Gisela Butera, Padma Sheila Rajagopal

**Affiliations:** National Cancer Institute; National Cancer Institute; National Cancer Institute; National Cancer Institute; National Institutes of Health Library; National Cancer Institute

## Abstract

**Purpose:**

Despite routine clinical collection of germline and somatic data in patients diagnosed with cancer, little is known about how these data concurrently associate with outcomes. The purpose of this review is to map the landscape of concurrent germline-somatic alterations and their associations with translational and clinical outcomes to identify addressable gaps in reporting and considerations for future research.

**Design:**

All studies in patients with cancer published through February 2024 were included that contained both germline and somatic data and associations of concurrent germline and somatic attributes with outcomes (e.g. *in silico*, predictive, prognostic, and clinical measures). Information abstracted from each study included publication date, study design, patient population characteristics, treatment regimen if applicable, germline data type, somatic data type, statistical interactions if performed, and reported associations.

**Results:**

Of the 8,613 studies screened, 197 met inclusion criteria. The most common concurrent germline-somatic alterations studied with respect to outcomes were germline *BRCA2*/somatic *TP53* and germline *BRCA1/*somatic *TP53* (n=40 and n=38 studies, respectively). Statistical testing was performed in 41.6% (n=82) of studies to determine associations between concurrent germline-somatic alterations and outcomes, with other studies providing solely descriptive or enumerative analysis. Among studies reporting direction of effect, 31 showed benefit associated with concurrent germline and somatic alterations relative to germline-only or somatic-only, while 28 showed harm.

**Conclusions:**

These findings suggest that planning for concurrent germline-somatic data analysis during the initial design of a translational or clinical study could meaningfully improve current applications in cancer genetics.

## Introduction

Over the past 10 years, germline (normal, inherited, or hereditary) and somatic (tumor-specific) molecular sequencing data are increasingly collected as part of standard-of-care management among individuals diagnosed with cancer. National Comprehensive Cancer Network^®^ (NCCN^®^) guidelines now recommend germline testing for patients in at least 15 cancer types, and somatic tumor testing in at least 11 cancer types.^1^ The Food and Drug Administration (FDA) has approved at least 43 treatment-selecting biomarkers derived from germline and somatic testing, including several that are “tumor agnostic” or applicable across tumor types.^2^ In 2020, the American Society for Clinical Oncology (ASCO) released a provisional clinical opinion recommending somatic testing for patients with advanced/metastatic cancer if there were potential biomarker-linked therapies available, with a concurrent recommendation to consider germline testing.^3^

Rapidly evolving recommendations together with the historical siloing of hereditary cancer genetics from clinical oncology can result in incomplete study design or non-aligned data collection of germline and somatic data. This limits the ability to research how concurrent germline and somatic data may inform cancer outcomes.^4^ In a 2022 study of oncology clinical trials, only 1.1% described collecting germline data. Within this small fraction of trials, *BRCA1 and/*or *BRCA2* mutation status alone were most often collected (~ 25%), while all other single gene or biomarker data were collected in less than 10% of trials doing so.^5^

This common limitation with existing data leaves a basic question unanswered: what are the predictive and prognostic implications of germline versus somatic origins of variants driving cancers? In characterizing loss of heterozygosity, Knudson originally described that in inherited retinoblastomas, people were born with an inherited “hit” in *RB1*, leaving them with only one functioning gene (loss of heterozygosity) that could become non-functional with another somatic “hit,” resulting in earlier cancers relative to non-inherited retinoblastomas requiring two such arbitrary somatic “hits.”^6^ Yet clinical behavior in cancers arising from germline versus somatic etiology differs even across genes for reasons that are not well understood. Treatment response patterns in cancers with either germline or somatic *BRCA1/2* mutations tend to be similar.^7,8^ However, cancers that acquire somatic *TP53* mutations (with no germline *TP53* alterations) have markedly more metastatic potential compared to cancers due to germline pathogenic variants in *TP53*.^9–12^

With no formal consensus as to how translational researchers or clinical oncologists should report concurrent germline and somatic data, studies may apply a variety of designs to clinical and genomic data that can be difficult to compare or translate. The issue of study design heterogeneity becomes amplified by the increasing popularity of real-world evidence studies, in which datasets must be harmonized for use.^13,14^

The objective of this systematic review, therefore, is to map the reported landscape of concurrent germline-somatic alterations and their associations with translational and clinical outcomes. We sought to characterize the most common forms of germline and somatic reporting, how associations are demonstrated, and the outcomes of greatest interest. We aimed to identify addressable gaps to optimize future research using genomic data in patients with cancer. Concurrent germline-somatic associations may have different associations with translational and clinical outcomes relative to associations observed when studying only germline or only somatic alterations. Our findings offer reasonable, high-yield ways to meaningfully address this question in future research.

## Methods

### Study design, inclusion/exclusion criteria, database search

Our study followed the Preferred Reporting Items for Systematic Reviews and Meta-analyses (PRISMA) 2020 guidelines and checklist and was prospectively registered in the PROSPERO database (CRD42022326884).^15^ The protocol was updated with minor edits during the systematic review process and the updates were documented in PROSPERO.

### Inclusion/exclusion criteria:

Studies were included if and only if they contained all the following:

- Human germline DNA sequencing data
- Human tumor DNA sequencing or other molecular data
- Association reported or evaluated statistically using both germline and somatic data as an independent variable and outcome as the dependent variable
- At least 10 patients with cancer of any age and any population

All studied outcomes were allowable, including *in silico* measures (e.g., mutational signatures), clinical measures (e.g., age of cancer onset, prognostic indicators), or clinical outcomes (e.g., treatment response, survival). Multiple study designs were acceptable, including non-randomized studies, given the lack of standardization of reporting formats in this space.

Studies were excluded if:

- Patients studied did not have any cancers, or the biological data were not of human origin
- If germline data were reported without somatic, if somatic data were reported without germline, or if no association was considered between concurrent germline and somatic data and an outcome
- If somatic data used emerging methods not yet widely applied in clinical practice (e.g. immune microenvironment, cell-free DNA, circulating tumor cells, exosomes)
- If search terms (Supplementary Methods) were discussed in the paper but without analysis
- Review articles or other articles that did not generate any new data

#### Database search:

A comprehensive search was conducted by a biomedical librarian (GB) from database inception using MEDLINE via PubMed, Embase, Cochrane Library, CINAHL, and Web of Science. We also performed a gray literature search in Google Scholar (first 300 records), reviewed clinical trials (in ClinicalTrials.gov and the World Health Organization’s International Clinical Trials Registry Platform), and conducted citation searching of reference lists of included studies. The search was performed without date limits or language restrictions. The original search was performed on June 15, 2022 and refreshed for additional articles on March 8, 2024. The complete search strategy and more details on exclusion criteria are available in Supplementary Methods.

### Study selection, data extraction and risk of bias

Citations identified through database searches were initially imported into EndNote reference management software (initially v.20, now v.21, Clarivate, Philadelphia, PA), where duplicates were removed, then imported into the current version of the Covidence screening software (Covidence systematic review software, Veritas Health Innovation, Melbourne, Australia). Covidence is a web-based screening tool that allows use of machine learning algorithms to streamline record screening by displaying citations based on users’ screening decisions that will be most likely to meet the eligibility criteria to improve time management and efficiency of the screening process. However, all citations were reviewed manually in addition to software recommendations. For each candidate study, two investigators among (LRP, AAS, JW, SA, PSR) voted initially on inclusion/exclusion after title/abstract and full-text screening. Any disagreements in the initial votes were reviewed subsequently among the voters and PSR to reach consensus. If two or more studies reported on the same patient population, the most recent was chosen.

### Data extraction and risk of bias assessment

A brief pilot of 25 articles was performed by three independent reviewers (LRP, AAS, PSR) to test data screening and extraction and to identify lacunae in the initial protocol; methods were refined based on the pilot. For studies meeting inclusion criteria, two independent investigators (among LRP, AAS, SA, JW, PSR) identified the relevant data source(s), such as a table or a figure. Having found the data source(s), the same two investigators extracted details including publication date, study design, characteristics of patient population under study, treatment regimen if applicable, germline data type, somatic data type, and use of any statistical testing.

*Study designs* were classified as exactly one among: case series, cross-sectional analyses, case-control studies, cohort studies, non-randomized and randomized interventional trials, and meta-analyses (if new measures were generated with individual-level data).

*Germline data* from each study were classified as being reported at the variant/single nucleotide polymorphism (SNP), polygenic score, gene, chromosomal region, mutational signature (such as microsatellite instability), or other type; data could be of multiple types. *Somatic data* from each study were classified as being from at least one of: variants, genes, copy-number alterations, mutational signatures, and other clinically relevant -omics data, including expression signatures, immunehistochemistry, loss of heterozygosity (LOH), methylation, or other.

*Associations* were described, with statistical test and output if statistically assessed. If not statistically assessed, data were collected from the paper as reported on a given outcome with respect to germline only, somatic only, or germline and somatic features.

*Outcomes* collected were characterized as clinical (age at diagnosis, stage, and other clinical features), predictive (response to treatment or known treatment-related biomarkers), prognostic (measures of survival), or *in silico* (calculated bioinformatic measures).

Investigators also performed a risk of bias assessment using the validated Prediction Model – Risk of Bias Assessment Tool (PROBAST), which applies to non-randomized and observational studies of biomarkers.^16^ Full details of data collection and quality assessment information including template are available in Supplementary Methods.

### Results reporting

Reporting in this systematic review is descriptive with limited statistical synthesis due to the significant heterogeneity of identified studies. Studies that performed statistical interaction and association testing with reporting are described using the vote counting method applied to the direction of effect. All statistical results were generated in R version 4.5.0 (R Core Team, 2025, Vienna, Austria) and the *robvis* Shiny app.^17^

## Results

### Concurrent Germline-Somatic Alterations and Associations with Cancer Outcomes

**Figure 1:**
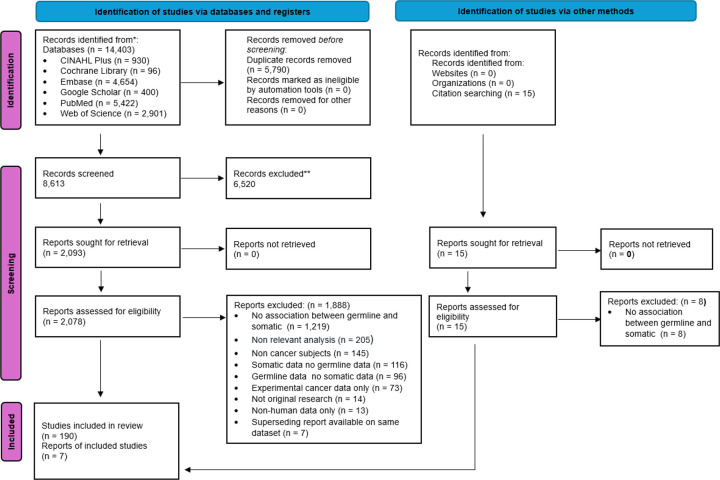
PRISMA flowchart of searched, screened, and included studies, as well as reasons for excluding studies. Source: Page MJ, et al. BMJ 2021;372: n71. doi: 10.1136/bmj.n71. This work is licensed under CC BY 4.0. To view a copy of this license, visit https://creativecommons.org/licenses/by/4.0/

This systematic review included 197 articles from among 8,613 articles screened at the title/abstract level and 2,093 articles screened at the full-text level (Fig. 1). Among articles excluded at the full-text level, over 1,200 (64.8%) were excluded solely because no association was measured between concurrent germline and somatic data and an outcome. In other words, these studies collected both germline and somatic data from patients with cancer but reported each data type in parallel only without any consideration of alterations concurrent in the same individual and outcome.

Characteristics of the 197 included articles are presented in Table 1. Included articles were published between 4/1997 and 2/2024, with the highest number (n = 32 studies, 16%) among calendar years being published in 2021. All included studies and streamlined extracted data are presented in **Supplementary Table S1.** All excluded studies are presented in **Supplementary Table S2**. The most common study designs were cross-sectional studies (n = 102, 52%) or cohort studies (n = 53, 27%). Among cancer types, studies were most often conducted on breast cancer (n = 45, 23%), multiple (at least 2+) cancer types (n = 27, 14%), or ovarian cancer (n = 20, 10%). Up to 25% of studies did not report standard clinical staging information, 49% of articles included patients who had previously received multiple lines of therapy in advance of data collection, and 58% of studies reported potential conflicts of interest. The median number of patients per study was 44 (range: 10 to 234,154 patients), and 16 studies were performed (at least in part) using data from The Cancer Genome Atlas (TCGA) and/or the Pan-Cancer Atlas of Whole Genomes (PCAWG). Study designs became larger, with more patients, inclusive of more cancer types, and more rigorous (extending to clinical trials) going into 2020 **(Supplementary Figure S1)**.

### Reporting of Germline Data, Somatic Data, Associations, and Cancer Outcomes

**Figure 2:**
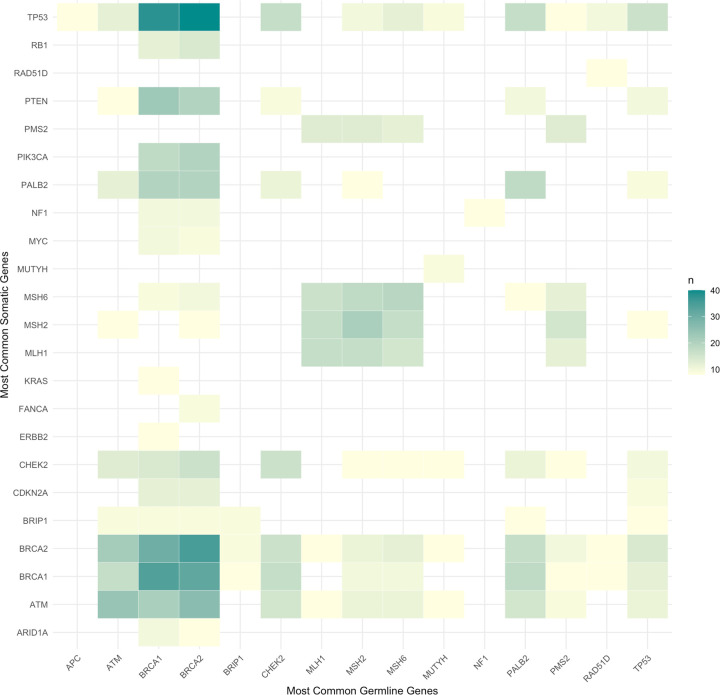
Heatmap of germline and somatic genes in most frequently observed pairings associated with an outcome. The X-axis includes the most common germline genes, and the Y-axis includes the most common somatic genes. The fewest studies are shaded in white, then yellow, with more studies shaded in green.

Germline data were reported at the gene level in 82% of studies, and somatic data at the gene level in 74% of studies. An additional 9% of studies evaluated gene-level associations and looked at other data types (e.g., variants, copy number alterations, etc.). (Table 2). We compared how often each germline/somatic data pair was evaluated across studies. Many studies evaluated multiple germline-somatic data pairs, and each pair was counted as 1 in this assessment without any normalization. The most frequent pairs are plotted by frequency in Fig. 2. The most common germline-somatic pairs evaluated across all studies (descriptively or statistically) were germline *BRCA2* and *BRCA1* with somatic *TP53* (n = 40 and n = 38 studies, 20% and 19% respectively). Germline and somatic *BRCA1* or *BRCA2* were the second most common concurrent pair (n = 30–35, 15–18%). After *BRCA1/*2, *ATM* was the most studied gene for concurrent germline and somatic alterations (n = 24, 12%). All concurrent germline-somatic alteration pairs and the number of studies for each are listed in **Supplementary Table S3.**

The most studied germline genes overall were *BRCA2* (in 974 data pairs), *BRCA1* (891 pairs), *TP53* (543 pairs), *ATM* (491 pairs), *CHEK2* (432 pairs), *PALB2* (403 pairs), *MSH2* (392 pairs), *MSH6* (382 pairs), *PMS2* (349 pairs) and *FANCA* (289 pairs). The most studied somatic genes overall were *TP53* (584 data pairs), *BRCA2* (484 pairs), *BRCA1* (389 pairs), *ATM* (370 pairs), *CHEK2* (334 pairs), *MSH2* (311 pairs), *PTEN* (281 pairs), *PALB2* (273 pairs), *BRIP1* (270 pairs), *MSH6* (249 pairs), *CDKN2A* (235 pairs), *MLH1* (201 pairs), *PIK3CA* (195 pairs), *PMS2* (186 pairs), and *FANCA* (182 pairs).

Statistical testing was performed in 42% (n = 82) of studies, with the remaining studies providing only numerical or descriptive data without a statistical assessment. The most common statistical tests among those with testing included Kaplan-Meier survival plots (n = 20 of 82, 24%), Cox proportional hazards (n = 17, 21%), and linear or logistic regressions (n = 10, 12%).

The most common cancer outcomes across all included studies were age at diagnosis (n = 19, 10%) and stage (n = 15, 8%) of clinical indications; treatment response (n = 24, 12%), tumor mutational burden (TMB) (n = 17, 9%) and homologous recombination deficiency (HRD) (n = 9, 5%) of predictive indicators, and progression-free survival (n = 16, 8%) and overall survival of prognostic indicators. Overall survival was the most studied outcome overall (n = 54, 28%). Within each outcome category, however, measurements varied (from binary variables to numerical ranges to specific scales), preventing further synthesis.

### Direction of Effect

**Figure 3:**
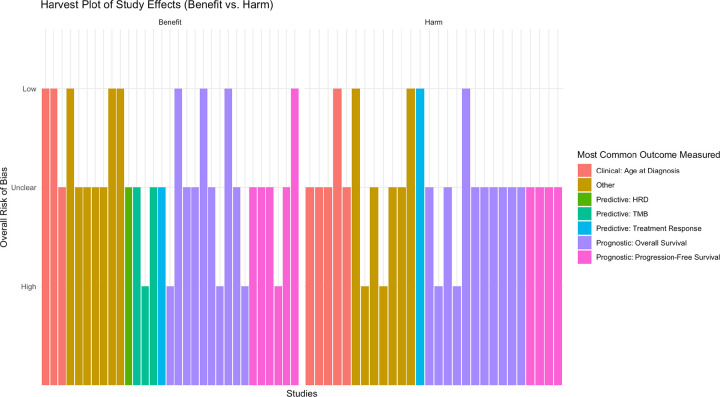
Harvest plot of vote-counted studies reporting benefit vs. harm. The X-axis includes the 59 studies that performed statistical testing and demonstrated that concurrent germline-somatic alterations were associated with better or worse outcomes in cancer relative to neither alteration/only one type of alteration. The Y-axis includes the overall risk of bias, with studies represented by higher bars having less risk of bias. We restricted studies with multiple outcomes to the most frequently studied outcomes, such that each study was represented once in the figure. HRD and TMB were used as outcomes in 4 studies and only showed evidence of benefit.

Given the heterogeneity across studies, which prevented further synthesis of outcomes data, we used vote counting based on direction of reported effect as recommended by the Cochrane Handbook to assess reported benefit vs. harm among studies that conducted any statistical analysis (Fig. 3).^18^ Benefits were those in which patients with cancer were reported by the study to have better clinical, predictive, or prognostic outcomes with concurrent germline-somatic alterations, and harms were those in which patients with cancer were found to have worse such clinical outcomes. Studies using *in silico* outcomes (where benefit vs. harm to patients could not be clearly characterized), and studies with only descriptive information, were accordingly not included in the direction of effect analysis. We observed that there were nearly equal frequencies for direction of effect between benefit and harm, with 31 studies showing benefit and 28 studies showing harm. Notably, the 4 studies with TMB or HRD as outcomes only reported benefits.

### Quality Assessment and Qualitative Risk of Bias

**Figure 4:**
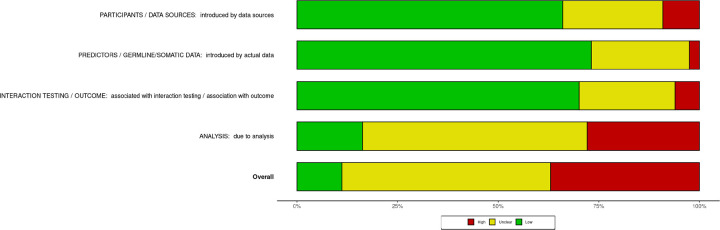
Weighted bar plot of the distribution of risk of bias judgments within each bias domain. The X-axis shows percent of studies. The Y-axis offers the different types of biases. Green is associated with low risk of bias, and red with high risk of bias.

Results of the PROBAST risk of bias assessment are detailed in Fig. 4. The most frequent source of bias was due to analysis performed, with the most common contributors being poor model performance measures (e.g., survival curves without censoring, no performance measures / prediction calculated) in 109 studies, no multivariate analysis performed in 157 studies, and no validation performed in 185 studies (**Supplementary Figure S2**). We cannot report publication bias due to lack of consistent reporting of standard errors.

## Discussion

This comprehensive systematic review offers the first overview of the landscape of concurrent germline-somatic alterations and associations with outcomes in patients with cancer, highlighting significant potential opportunities for researchers seeking to study co-occurring germline-somatic alterations.

We were encouraged by our identification of shared starting points for harmonizing concurrent germline and somatic data that have naturally occurred in the literature. Both germline (82%) and somatic (74%) data were reported at the gene level (as opposed to the variant level or more complex structural variants). Predictive outcomes (32%) and prognostic outcomes (43%) are already represented. Since 2020, there has been a trend towards larger studies, more patients, and greater analytical rigor.

Our harvest plot illustrates the need to study concurrent germline-somatic alterations. Approximately half of the studies with statistical analyses and patient-related benefit/harm outcomes showed beneficial outcomes associated with concurrent germline-somatic variants, and half showed harmful outcomes. Clarification of when patients with concurrent germline-somatic variants in their cancers may experience benefits vs. harms is an essential, clinically relevant question. We note that the concepts of “benefit” and “harm,” while limited in this manuscript to specific quantitative clinical outcomes, absolutely invite complex interpretations outside the scope of this manuscript but worth evaluating in the context of treatment options and patient experience.

Our systematic review curated data that can be used as a foundation for future research. Supplementary Table S1 provides detailed information on each of the included studies based on cancer type, number of patients, germline and somatic data, outcome measures and statistical testing or descriptive measures. It is intended to be a starting reference for translational and clinical researchers interested in concurrent germline-somatic alterations and associations with outcomes.

Our findings also highlight potential high-yield interventions to address common challenges that could improve translational and clinical applicability of genomic data. One of the primary reasons for nearly 65% of articles being excluded during the full-text step is easily addressable: No evaluation of association between concurrent germline-somatic alterations and outcome. Per our protocol, to be excluded for this reason meant that there were no other reasons. We frequently observed “near miss” studies with detailed germline and somatic data reported in parallel, but no way to align this data for the study subjects even when cancer outcomes were available (as observed in the ARIEL3 (NCT01968213) or PROfound (NCT02987543) publications, for example).^20,21^ We also observed studies that, by design, sought to characterize germline contribution to somatic data and to outcomes without using the germline data predictively or prognostically.^22–26^ Supplementary tables that provide even descriptive data for germline-only, somatic-only, and germline-somatic with outcomes of interest would meaningfully add to our existing knowledge.

Analytic limitations, as a major issue, were highlighted by our PROBAST risk of bias assessment, with most studies lacking any multivariate analysis or validation. Consideration of statistical testing or validation datasets to support any co-occurring germline-somatic alterations associated with outcomes, if possible, would be another meaningful step towards increasing potential clinical applicability.

Included studies focused heavily on genes associated with rare hereditary cancer predisposition syndromes. This is understandable given that LOH classically serves as an explicit, well-studied mechanism of germline-somatic interaction.^6,19^ Ongoing focus on LOH in the same known 20–30 genes and handful of cancer types leaves wide open the chance to study emerging somatic biomarkers and their co-occurrence with germline genes of interest as related to outcomes. For example, it is not clear how concurrent germline *BRCA1/2* variants and somatic *PIK3CA* targetable alterations may be associated with differences in response to alpelisib if at all relative to somatic *PIK3CA* targetable alterations only without germline *BRCA1/2* variants.

Another major challenge of studying concurrent germline-somatic alterations, particularly for rare pathogenic variants, is statistical power. Even among the 197 studies that met our inclusion criteria, basic clinical staging information was missing in up to 25%, and treatment data were heterogeneous (an obvious confounder) in nearly 50%. Anticipatory planning for concurrent germline-somatic associations with outcome would allow translational and clinical researchers to obtain funding and develop analytic plans to intentionally compare germline-only, somatic-only, or concurrent germline-somatic data. Such synthesis of germline and somatic data can, for example, facilitate clearer understanding of how respective concurrent germline and somatic contributions are associated with more frequent occurrence of TMB or HRD.

We did not seek to study germline → outcome^27–29^, somatic → outcome^30–33^, or germline → somatic^34,35^ relationships in this systematic review given its already large scope. Many excellent efforts describing those types of (bio)logical relationships have been published within cancer types. One important caveat of our findings is that such germline-somatic relationships as reviewed may represent passenger or driver relationships underlying observed associations in these studies.

Given the widespread, increasing use of germline and somatic testing in oncology for predictive and prognostic information, translational and clinical cancer genetics researchers owe it to patients to use their data to guide either their care or the care of future patients as optimally, fully, and accurately as possible. From a clinical standpoint, physicians currently remain unable to counsel patients on how co-occurring germline and somatic biomarkers predict treatment response or prognosis. This systematic review sketches the landscape of this phenomenon and outlines paths to improve current clinical and translational reporting of cancer genetics data to answer such questions.

## Supplementary Files

This is a list of supplementary files associated with this preprint. Click to download.

- 2025.9.11GSSuppMethods.docx
- GSSupplementaryTableS123.xlsx
- S1.pdf
- S2.pdf

## Figures and Tables

**Table 1:** Descriptive information about studies, including study design, cancer types included, stages included, treatment categories included, whether the authors had conflicts of interest, and if the initial data collection was performed using The Cancer Genome Atlas (TCGA) or Pan-Cancer Analysis of Whole Genomes (PCAWG).

	N = 197	%
**Study Design**		
Cross-Sectional Study	102	52%
Cohort	53	27%
Cases	16	8%
Non-randomized interventional trial	15	8%
Randomized controlled trial	8	4%
Case-Control	2	1%
Meta-analysis	1	1%
**Cancer Types**		
Adrenocortical	1	1%
Brain	7	4%
Breast	45	23%
Breast (Lobular) = 1		
Breast (Male) = 2		
Breast (TNBC) = 7		
Colon	13	7%
Cutaneous T-cell Lymphoma	2	1%
Esophageal Cancer	2	1%
GIST	1	1%
Hepatocellular Carcinoma	1	1%
Head and Neck Cancer	6	3%
Hematologic Malignancies (Not Specified)	11	6%
Lung Cancer (NSCLC/SCLC)	5	3%
Lymphoid Neoplasms	1	1%
Malignant Peripheral Nerve Sheath Tumors	1	1%
Melanoma	4	2%
Mesothelioma	2	1%
Myeloid Neoplasms	10	5%
Ovarian Cancer	20	10%
Pan-Cancer	27	14%
Pancreatic Cancer	10	5%
Pancreatic Neuroendocrine Tumor	1	1%
Papillary Thyroid	1	1%
Pheochromocytoma / Paraganglioma	2	1%
Pituitary Blastoma	1	1%
Prostate Cancer	11	6%
Renal Cell Cancer	3	2%
Retinoblastoma	2	1%
Sertoli-Leydig Cell Tumors	1	1%
Urothelial Cancer	1	1%
Uterine Cancer	2	1%
Wilms Tumor	3	2%
**Stages Included**		
Early-Stage	25	13%
Advanced / Metastatic	41	21%
All	79	40%
Other	24	12%
Not Applicable	12	6%
Unknown	16	8%
**Treatments Studied**		
Heterogeneous	97	49%
Targeted Therapy	16	8%
Chemotherapy	15	8%
Mixed	6	3%
Immunotherapy	5	3%
Hormone Therapy	1	1%
Surgery	3	2%
Transplant	1	1%
None Reported	53	27%
**Conflicts of Interest**		
Yes	114	58%
**Was this Study Performed in TCGA or PCAWG?**		
TCGA	15	8%
PCAWG	1	1%

**Table 2:** Information about data included on studies, including germline data, somatic data, and the most common cancer outcomes studied.

	N = 197	%
**Germline Data**		
Genes	162	82%
Variants/SNPs	26	13%
Mutational Signatures	3	2%
Polygenic Scores	2	1%
Chromosomal Regions	1	1%
Other	3	2%
**Somatic Data**		
Genes	146	74%
Genes AND		
Chromosomal Regions	4	2%
Expression Signatures	1	1%
Immunohistochemistry	1	1%
Methylation	1	1%
Mutational Signatures	4	2%
Somatic Copy-Number Alterations	4	2%
Variants	8	4%
Variants AND Methylation	1	1%
LOH	10	5%
Mutational Signatures	7	4%
Somatic Copy-Number Alterations	2	1%
Immunohistochemistry	1	1%
Other	7	4%
**Most Common Cancer Outcomes Studied**		
Clinical: Age at Diagnosis	18	9%
Clinical: Stage	17	9%
Predictive: Treatment Response	15	8%
Predictive: TMB	17	9%
Predictive: HRD	9	5%
Prognostic: Progression-Free Survival	15	8%
Prognostic: Overall Survival	55	28%
Other		

## Data Availability

Original data sharing is not applicable to this article as no individual-level data were obtained or analyzed in this study. The data underlying this article are available in the article and in its online supplementary material.
